# Frequency and Correlates of Online Consultations With Doctors or Therapists in Middle-Aged and Older Adults: Nationally Representative Cross-sectional Study

**DOI:** 10.2196/29781

**Published:** 2022-04-07

**Authors:** André Hajek, Hans-Helmut König

**Affiliations:** 1 Department of Health Economics and Health Services Research University Medical Center Hamburg-Eppendorf Hamburg Germany

**Keywords:** online consultations, doctor, therapists, telehealth, COVID-19, SARS-CoV-2, digital health

## Abstract

**Background:**

A few studies have identified the frequency and correlates of online consultations with doctors or therapists. However, there is a lack of studies using nationally representative data from middle-aged and older adults in Germany.

**Objective:**

This study aims to determine the frequency and correlates of online consultations with doctors or therapists in Germany.

**Methods:**

For this study, cross-sectional data were taken from the nationally representative German Ageing Survey (DEAS; n=3067 in the analytical sample; age range 46-98 years). As part of the DEAS, a short survey was conducted between June 8 and July 22, 2020, examining the everyday life and living conditions among these middle-aged and older individuals during the COVID-19 pandemic. The frequency of online consultations with doctors or therapists served as the dependent variable (daily, several times a week, once a week, 1-3 times a month, less often, and never). Multiple logistic regressions were performed.

**Results:**

In sum, 10.02% (381/3806) of individuals with access to the internet had online consultations with doctors or therapists. Multiple logistic regressions showed that the likelihood of using online consultations with doctors or therapists (compared with those never using such services) was positively associated with higher education (compared with medium education; odds ratio [OR] 1.31, 95% CI 1.01-1.70), living with a partner in the same household (compared with single; OR 1.53, 95% CI 1.05-2.22), poorer self-rated health (OR 1.42, 95% CI 1.16-1.74), increased loneliness (OR 1.45, 95% CI 1.10-1.90), and increased satisfaction with life (OR 1.30, 95% CI 1.03-1.64).

**Conclusions:**

Study findings suggest that a non-negligible proportion of about 1 out of 10 individuals aged 46 years and over had online consultations with doctors or therapists. However, compared with other countries, this proportion remains small. Knowledge about the correlates of (non)use may assist in identifying corresponding individuals. In times of reshaping the health care system, these efforts in online consultations with doctors or therapists may contribute to addressing patient needs. Moreover, increased use of such services may reduce the risk of getting infected with SARS-CoV-2 by reducing social contact.

## Introduction

In the last 2 decades, with better access to the internet, and more high-speed connections, online consultations with doctors or therapists have grown continually [1]. Online consultations can improve patient outcomes via increased access to care and medical information [1]. Furthermore, online consultations can increase job satisfaction and work-life balance among physicians [2]. Moreover, there is an increased need for online consultations due to increased economic costs, poor access in rural areas, and demographic aging, with an increasing number of individuals in old age suffering from various chronic illnesses.

During the COVID-19 pandemic, the use of online consultations sought to reduce unnecessary social contact by reducing the number of in-person consultations [3]. This can reduce the risk of getting infected with SARS-CoV-2, which is key among older adults with risk factors for more severe experiences of COVID-19 [4].

Online consultations with doctors or therapists can, for example, be used for issuing sick notes or care of patients after surgery. A recent systematic review recommended use of such consultations in orthopedics [5]. Additionally, it has been shown that patient satisfaction with video consultations was high to very high during the COVID-19 pandemic [6].

It should be noted that the term doctor (*arzt*) in Germany refers to physicians, while the term therapists (*therapeut*) commonly refers to psychological or medical psychotherapists, as well as other therapists (eg, occupational therapists or physical therapists). According to the German Medical Association, the total number of currently working physicians (registered with state medical associations) was around 409,000 in 2020 [7]. The physician density (inhabitants per working physician) varies between the states in Germany—from 133 (Hamburg) to 248 (Brandenburg) [7]. In Germany, around 12,000 specialists worked in psychiatry and psychotherapy in 2020 [8] and around 48,000 psychological psychotherapists worked in 2019 [9]. Moreover, around 203,000 individuals work as physical therapists in Germany (data from 2019) [10] and around 59,000 individuals work as occupational therapists (data from 2017) [11]. Regional differences are present (eg, between rural and urban areas and between certain states) [10].

It may be worth describing online consultations with doctors or therapists in Germany. Certain video consultations have been included in the outpatient medical fee schedule (EBM) applied for patients who are members of a statutory health insurance (approximately 90% of the population) in Germany since April 2017. Moreover, members of a private health insurance (around 10% of the population) can use online consultation as it is in accordance with the medical fee schedule used for privately insured patients (GOÄ). According to the National Association of Statutory Health Insurance Physicians (KBV) [12], around 25% of doctors’ offices offered video consultations in Germany in March 2020, whereas only 2% offered such services in 2017. Furthermore, around 3000 hours of online consultations were conducted in 2019, whereas 1.4 million hours of online consultations were conducted in the first half of 2020 [12]. Besides, 3 out of 4 online consultations were conducted in the field of psychotherapy in the first half of 2020. Additionally, around 12% of the general practitioners (GPs) offered online consultations in the second quarter of 2020 [12]. Online consultations are valued by GPs, medical specialists, and hospital doctors in Germany [13]. However, it is agreed that they cannot substitute care in various specialties, such as hand surgery, but rather complement care [14].

In Germany, there is still a general lack of data on the frequency of online consultations with doctors or therapists based on *nationally representative samples* of middle-aged and older adults. Because of this lack in knowledge, our aim was to determine the frequency and correlates of online consultations with doctors or therapists *based on nationally representative data from Germany*. We would like to note that identifying the correlates of such online consultations was an explorative aim of this study (without any prespecified hypotheses). Knowledge about the use of online consultations is important for policy makers. For example, based on this knowledge, strategic planning could be established and implemented to identify and support nonadopters of online consultations. This could assist in improving the uptake of online consultations.

## Methods

### Sample

Data for this study were taken from the German Ageing Survey (DEAS; a nationally representative sample of individuals aged 40 years in Germany). As part of the DEAS, a short survey was conducted between June 8 and July 22, 2020, examining the everyday life and living conditions among these middle-aged and older individuals during the COVID-19 pandemic. This survey was addressed to all panel participants who had taken part in previous DEAS waves at least once. Thus, the basis for this survey was all willing panel participants who could still be reached from the baseline samples 1996 to 2014 (from 1996: 539 individuals; from 2002: 525 individuals; from 2008: 1549 individuals; from 2014: 2210 individuals). Thus, individuals were at least 46 years old in the survey conducted in 2020.

In sum, 4823 individuals who had already participated in 1 or more former waves were included in this short survey (paper and pencil interview) with a response rate of 56.5%, which is comparable to other survey studies in Germany [15]. Of the included sample, 3806 individuals had access to the internet and filled out the dependent variable (analytical sample in regressions analysis: n=3067; reduction in sample size can be explained by missing data in the independent variables, as listwise deletion was used in regression analysis [16]). Further details regarding the DEAS study are given elsewhere [17].

### Dependent Variables

Individuals with access to the internet (ie, individuals who reported “yes” when asked whether he or she has access to the internet) were asked to report the frequency of “consultations with doctors or therapists via an online platform” (daily, several times a week, once a week, 1-3 times a month, less often, and never). It was dichotomized (0=never; 1=otherwise including daily, several times a week, once a week, 1-3 times a month, and less often). This variable served as the dependent variable.

### Independent Variables

We included socioeconomic, lifestyle-related, health-related, COVID-19–related factors, and psychosocial factors as independent variables. With regard to socioeconomic factors, we included age, sex, educational level (International Standard Classification of Education 97 [ISCED-97] [18]: low, medium, or high education), employment status (employed, retired, non-employed), living situation (single, with partner in household, with partner without a common household), having at least one child (no or yes), migration background (no or yes), monthly household net income (in Euros), region (West Germany or East Germany), and type of district (large cities, urban cities, urban-rural districts, rural districts).

With regard to lifestyle factors, we included engagement in physical activities and the frequency of walks (in both cases: daily, several times a week, once a week, 1-3 times a month, less often, never). With regard to health-related factors, we included self-rated health (from 1=very good to 5=very bad) and depressive symptoms (10-item Center for Epidemiological Studies-Depression [CES-D] scale [19] score ranging from 0 to 30, with higher scores reflecting more depressive symptoms). In this study, Cronbach alpha for the CES-D was .85. The CES-D has favorable psychometric characteristics [20].

With regard to COVID-19–related factors, we included the following variables: feeling that the COVID-19 crisis posed a threat to oneself (from 0=not at all a threat for me to 10=extreme threat for me); infection among people in one’s own personal environment with the coronavirus (yes, no, don’t know); one’s own infection with the coronavirus (yes, no, don’t know); and the feeling that one can influence an infection with the coronavirus (from 1=not at all to 7=entirely). With regard to psychosocial factors, we included life satisfaction (Satisfaction with Life Scale [SWLS] by Diener et al [21], which includes 5 items, scored from 1 to 5, where higher values correspond to greater satisfaction with life) and loneliness (6-item De Jong Gierveld Loneliness Scale [22]; scored from 1 to 4, with higher values reflecting higher loneliness levels). In this study, Cronbach alpha for the SWLS was .87 and that for the De Jong Gierveld Loneliness Scale was .79. Both tools (loneliness [23] and life satisfaction [24]) have favorable psychometric properties.

### Statistical Analysis

In a first step, sample characteristics were computed, stratified by the use of online consultations with doctors or therapists (unpaired *t* tests and chi-square tests were used, as appropriate). Subsequently, multiple logistic regressions were conducted to identify the correlates of use of online consultations with doctors or therapists. Statistical significance was set at *P*<.05. Stata 16.1 (StataCorp) was used to perform statistical analyses.

### Ethics Approval

Written informed consent was obtained from all individual participants included in the study. An ethics vote was not deemed necessary according to criteria for the need of an ethical statement. This is in line with the German Research Foundation guidelines. All procedures performed in studies involving human participants were in accordance with the ethical standards of the institutional or national research committee and with the 1964 Helsinki declaration and its later amendments or comparable ethical standards.

## Results

### Description of the Sample

Stratified by the use of online consultations with doctors or therapists, sample characteristics are presented in Table 1. In the total sample, the average age was 67.6 years (SD 9.7; range 46-98 years) and 49.31% (1877/3806) of the individuals were female. Overall, 10.02% (381/3806) of the individuals with access to the internet had online consultations with doctors or therapists.

In bivariate analysis, individuals using online consultations with doctors or therapists significantly (at least *P*<.05) differed from those who had never had an online consultation in terms of sex, living situation, presence of children, self-rated health, depressive symptoms, feeling that the COVID-19 crisis poses a threat to oneself, and loneliness. Further details are given in Table 1.

**Table 1:** Sample characteristics stratified by the use of online consultations with doctors or therapists (German Ageing Survey, fifth wave, n=3806).

Characteristics		Total (n=3806)^a^	Never had online consultations with doctors or therapists (n=3425)^a^	Have had online consultations with doctors or therapists (n=381)^a^	*P* value^b^
**Sex**					.03
	Men	1929	1716 (88.96)	213 (11.04)	
	Women	1877	1709 (91.05)	168 (8.95)	
Age (years), mean (SD)		67.6 (9.7)	67.6 (9.6)	68.0 (10.2)	.37
**Educational level (ISCED-97^c^ classification)**					.10
	Low (ISCED 0-2)	104	90 (86.54)	14 (13.46)	
	Medium (ISCED 3-4)	1687	1536 (91.05)	151 (8.95)	
	High (ISCED 5-6)	2014	1798 (89.28)	216 (10.72)	
**Employment status**					.78
	Employed	1196	1083 (90.55)	113 (9.45)	
	Retired	2412	2166 (89.80)	246 (10.20)	
	Non-employed	143	129 (90.21)	14 (9.79)	
**Living situation**					.01
	Single	737	685 (92.94)	52 (7.06)	
	With partner in the same household	2851	2548 (89.37)	303 (10.63)	
	With partner without a common household	168	149 (88.69)	19 (11.31)	
**Having at least one child**					.006
	No	321	303 (94.39)	18 (5.61)	
	Yes	3311	2964 (89.52)	347 (10.48)	
**Migration background**					.11
	No	3623	3267 (90.17)	356 (9.83)	
	Yes	177	153 (86.44)	24 (13.56)	
Household net income, mean (SD)		4175.8 (12,363.9)	4152.6 (12,778.0)	4384.9 (7690.7)	.74
**Region**					.21
	West Germany	2742	2457 (89.61)	285 (10.39)	
	East Germany	1064	968 (90.98)	96 (9.02)	
**Type of district**					.92
	Large cities	1058	954 (90.17)	104 (9.83)	
	Urban cities	1438	1293 (89.92)	145 (10.08)	
	Urban-rural cities	770	696 (90.39)	74 (9.61)	
	Rural districts	540	482 (89.26)	58 (10.74)	
**Engagement in physical activities**					.41
	Daily	454	407 (89.65)	47 (10.35)	
	Several times a week	1395	1244 (89.18)	151 (10.82)	
	Once a week	703	629 (89.47)	74 (10.53)	
	1-3 times a month	253	230 (90.91)	23 (9.09)	
	Less often	676	613 (90.68)	63 (9.32)	
	Never	303	282 (93.07)	21 (6.93)	
**Frequency of walks**					.33
	Daily	712	634 (89.04)	78 (10.96)	
	Several times a week	1447	1292 (89.29)	155 (10.71)	
	Once a week	651	598 (91.86)	53 (8.14)	
	1-3 times a month	277	251 (90.61)	26 (9.39)	
	Less often	572	514 (89.86)	58 (10.14)	
	Never	122	114 (93.44)	8 (6.56)	
Self-rated health (scored from 1=very good to 5=very bad), mean (SD)		2.4 (0.8)	2.4 (0.8)	2.6 (0.8)	<.001
Depressive symptoms (10-item Center for Epidemiological Studies-Depression scale; scored from 0 to 30, with higher values reflecting more depressive symptoms), mean (SD)		8.2 (4.7)	8.1 (4.7)	9.2 (5.1)	<.001
Feeling that the COVID-19 crisis is a threat for oneself (scored from 0=not at all a threat for me to 10=extreme threat for me), mean (SD)		4.0 (2.1)	4.0 (2.1)	4.3 (2.3)	.02
**Infection among people in one’s own personal environment with coronavirus**					.19
	Yes	307	272 (88.60)	35 (11.40)	
	No	3345	3022 (90.34)	323 (9.66)	
	Don’t know	129	111 (86.05)	18 (13.95)	
**Own infection with the coronavirus**					.79
	Yes	17	15 (88.24)	2 (11.76)	
	No	3547	3195 (90.08)	352 (9.92)	
	Don’t know	213	189 (88.73)	24 (11.27)	
Feeling that I can influence an infection with the coronavirus (scored from 1=not at all to 7=entirely), mean (SD)		4.6 (1.4)	4.6 (1.4)	4.5 (1.5)	.31
Loneliness (6-item De Jong Gierveld loneliness scale; scored from 1 to 4, with higher values reflecting higher loneliness levels), mean (SD)		1.9 (0.5)	1.9 (0.5)	2.0 (0.5)	<.001
Life satisfaction (Satisfaction with Life Scale; scored from 1 to 5, with higher values corresponding to greater satisfaction with life), mean (SD)		3.9 (0.7)	3.9 (0.7)	3.9 (0.7)	.67

^a^Data are presented as n or n (%) unless stated otherwise.

^b^*P* values were based on independent unpaired *t* tests and chi-square tests, as appropriate.

^c^ISCED: International Standard Classification of Education 97.

### Regression Analysis

The findings of multiple logistic regressions with experience of an online consultation(s) with doctors or therapists as the outcome measure are shown in Table 2. Multiple logistic regressions showed that the likelihood of having online consultations with doctors or therapists (compared with never using such services) was positively associated with higher education (compared with medium education; odds ratio [OR] 1.31, 95% CI 1.01-1.70), living with a partner in the same household (compared with single; OR 1.53, 95% CI 1.05-2.22), poorer self-rated health (OR 1.42, 95% CI 1.16-1.74), increased loneliness (OR 1.45, 95% CI 1.10-1.90), and increased satisfaction with life (OR 1.30, 95% CI 1.03-1.64). Other socioeconomic, lifestyle-related, health-related, and COVID-19–related factors did not achieve statistical significance (*P*>.05).

**Table 2:** Determinants of online consultations with doctors or therapists. Results of multiple logistic regression analysis (German Ageing Survey, short survey).^a^

Independent variables		Online consultations with doctors or therapists, OR (95% CI)
Sex: Women (reference: men)		0.86 (0.66-1.11)
Age		1.01 (0.99-1.03)
**Educational level (ISCED-97^b^ classification)**		
	Low education (reference: medium education)	1.39 (0.66-2.92)
	High education	1.31^c^ (1.01-1.70)
**Employment status**		
	Retired (reference: employed)	0.83 (0.55-1.24)
	Non-employed	0.88 (0.44-1.76)
**Living situation**		
	With partner in the same household (reference: single)	1.53^c^ (1.05-2.22)
	With partner without a common household	1.52 (0.79-2.93)
Having at least one child: Yes (reference: no)		1.58^d^ (0.95-2.63)
Migration background: Yes (reference: no)		1.11 (0.63-1.95)
Household net income (in €1000)^e^		1.00 (0.99-1.01)
Region: East Germany (reference: West Germany)		0.88 (0.64-1.21)
**Type of district**		
	Large cities (reference: rural districts)	0.84 (0.56-1.26)
	Urban cities	0.77 (0.51-1.16)
	Urban-rural cities	0.79 (0.52-1.20)
**Engagement in physical activities**		
	Several times a week (reference: daily)	1.22 (0.79-1.87)
	Once a week	1.17 (0.72-1.88)
	1-3 times a month	0.87 (0.47-1.64)
	Less often	0.89 (0.54-1.48)
	Never	0.59 (0.30-1.18)
**Frequency of walks**		
	Several times a week (reference: daily)	1.01 (0.71-1.44)
	Once a week	0.81 (0.52-1.25)
	1-3 times a month	0.87 (0.50-1.52)
	Less often	1.12 (0.72-1.73)
	Never	0.73 (0.29-1.82)
Self-rated health (from 1=very good to 5=very bad)		1.42^f^ (1.16-1.74)
Depressive symptoms (10-item Center for Epidemiological Studies-Depression scale, from 0 to 30, with higher values reflecting more depressive symptoms)		1.02 (0.99-1.06)
Feeling that the COVID-19 crisis poses a threat to oneself (from 0=not at all a threat for me to 10=extreme threat for me)		1.03 (0.97-1.09)
**Infection among people in one’s own personal environment with coronavirus**		
	No (reference: yes)	0.86 (0.56-1.31)
	Don’t know	1.57 (0.78-3.18)
**Personal experience of infection with the coronavirus**		
	No (reference: yes)	0.08 (0.00-1.73)
	Don’t know	0.07^d^ (0.00-1.59)
Feeling that one can influence an infection with the coronavirus (from 1=not at all to 7=entirely)		0.97 (0.89-1.07)
Loneliness (6-item De Jong Gierveld loneliness scale; from 1 to 4, with higher values reflecting higher loneliness levels)		1.45^g^ (1.10-1.90)
Life satisfaction (Satisfaction with Life Scale; from 1 to 5, with higher values corresponding to greater satisfaction with life)		1.30^c^ (1.03-1.64)
Constant		0.04^d^ (0.00-1.52)
Observations		3067
Pseudo R^2^		0.04

^a^Missing values were handled using listwise deletion. Outcome: 0=never using online consultations with doctors or therapists; 1=using online consultations with doctors or therapists.

^b^ISCED-97: International Standard Classification of Education 97.

^c^*P*<.05.

^d^*P*<.10.

^e^€1 = US $1.10.

^f^*P*<.001.

^g^*P*<.01.

## Discussion

### Principal Findings

In sum, 10.02% (381/3806) of the individuals with access to the internet had used online consultations with doctors or therapists. Regressions showed that the likelihood of having used online consultations with doctors or therapists (compared with never using such services) was positively associated with higher education, living with partner in the same household (compared with single), poorer self-rated health, increased loneliness, and increased satisfaction with life.

### Previous Research and Possible Explanations

To date, only a few international studies have reported the frequency of online consultations with doctors or therapists *based on nationally representative samples*. For example, 46.1% of GP services were provided using such consultations (video and telephone) in Australia in early May 2020 [25]. Moreover, 44% of respondents supported using online consultations for medication abortion among adults in the United States during the COVID-19 pandemic [26]. In general, the quite high proportion of individuals using online consultations with doctors or therapists (compared with the time prior to the pandemic in Germany) found in our study is in accordance with recently conducted research covering the adult population in Germany during the pandemic [27]. Because of the high proportion of online consultations, we assume that, in most cases, online consultations replaced physical meetings. However, because these data were not available in the data set used in our study, future research is required to clearly distinguish between different types of online consultations with doctors or therapists (ie, replacing or complementary to in-person consultations).

This high proportion supports the conclusion made by Wosik et al [28], who stressed the rise of virtual care. In international comparison (eg, compared with other European countries), Germany still lags behind [29]. By contrast, consumer-enabled and connected health technologies are widespread in the Netherland and Nordic countries.

To date, some studies have described the frequency and correlates of online consultations with doctors or therapists. For example, it has recently been shown that older, female, or poorer patients had used video consultations less frequently [30] in the United States. A greater reluctance to use online consultations with doctors was also reported among individuals with a lower educational attainment in Denmark [31].

Given that individuals filled out the questionnaire in June or July 2020, it appears plausible that those with poorer self-rated health had a greater likelihood of using online consultations, compared with those who did not use online consultations, as these individuals were in need of care and around 1.4 million hours of online consultations were made in Germany during the first half of 2020 [12].

Compared with single individuals, individuals living with a partner in the same household had a greater likelihood of using online consultations. This might be explained by the fact that partners may urge the individuals to use such services [32] when individuals are in need of care. Moreover, they could assist when technical difficulties arise. Furthermore, they may seek to protect their partner from possible infection with SARS-CoV-2.

The association between higher education and an increased likelihood of using online consultations appears to be plausible. This is due to the fact that higher education is often associated with lower computer anxiety and better computer skills [33].

With regard to other correlates, it was particularly surprising that COVID-19–related factors were not significantly associated with the outcome measure. It seems that other factors (ie, education, health, and psychosocial factors) are important for the outcome measure. Future research is required to clarify the association between COVID-19–related factors and the use of online consultations with doctors or therapists.

At first glance, it may seem contradictory that both higher loneliness and greater life satisfaction are associated with a greater likelihood of using online consultations. However, previous research has also demonstrated a link between increased loneliness and an increased number of GP visits [34]—for example, to address social needs in higher age [35]. Furthermore, the association between life satisfaction and online consultations may be explained by the fact that life satisfaction is positively associated with an increased use of preventive health care services (eg, in women: higher likelihood of obtaining a mammogram, x-ray, or pap smear; in men: higher likelihood of obtaining a prostate examination) [36]. Additionally, higher life satisfaction is associated with higher meaning in life (ie, a sense of comprehension and significance in life [37]) [38]. In turn, a higher meaning in life [39] is associated with more frequent health care use (GP and specialist visits) and an increased use of preventive health care services [40]. Individuals with high life satisfaction and high meaning in life may particularly value their lives and may use health care services (curative and preventive) to stay healthy for as long as possible [40].

### Strengths and Limitations

Some strengths and limitations of this study should be considered. This is the first study identifying the frequency and correlates of online consultations with doctors or therapists among individuals in the second half of life in Germany. Additionally, a nationally representative sample (data collection during the pandemic) was used. While the outcome measure had a high face validity, future studies are needed to distinguish between online consultations with doctors (including the medical specialty) or therapists (eg, psychotherapists, occupational therapists, or physical therapists). For example, it may be the case that online consultations with doctors differ from online consultation with therapists (eg, in terms of the reason for the consultation, the objective, and the average duration). Online consultations may also differ between, for example, physicians and psychological or medical psychotherapists. Differences may also exist between occupational therapists and physical therapists regarding online consultations. Moreover, other domains of health care use (eg, outpatient physician visits or hospital stays) and other health-related factors (eg, chronic conditions) were not assessed in this short survey. A small sample selection bias has been identified in the DEAS study [41]. However, the distribution of family status, family composition, labor force participation, and educational level is very close to the distribution in the German population [41]. Furthermore, this study examined patient-related factors, whereas more studies are required to investigate physician-related factors such as practice size [42] or region (rural vs urban [43]) when identifying the correlates of online consultations with doctors or therapists.

### Conclusions

Findings of this study suggest that a non-negligible proportion of around 1 in 10 individuals aged 46 years and over had online consultations with doctors or therapists. However, compared with other countries, there is still room for improvement (regarding the proportion of online consultations by community-dwelling individuals aged 46 years and over in Germany). Examination and comparison of the characteristics of adopters and nonadopters of online consultations could assist in strategic planning and improve uptake of online consultation. Future research in other countries is required.

With regard to Germany, it may be beneficial to strongly intensify efforts linked to broadband infrastructure in Germany, which has clear potential for improvement and still lags behind other countries. A good quality and stable connection, as well as perhaps monetary incentives for both patients (eg, cost savings) and doctors/therapists (eg, remuneration incentives) may assist in increasing the proportion of online consultations. Furthermore, strategies to reduce computer anxiety, particularly among the oldest old, may assist in this.

## Abbreviations

CES-D: Center for Epidemiological Studies-Depression
DEAS: German Ageing Survey
EBM: outpatient medical fee schedule
GOÄ: medical fee schedule used for privately insured patients
GP: general practitioner
ISCED-97: International Standard Classification of Education 97
KBV: National Association of Statutory Health Insurance Physicians
OR: odds ratio
SWLS: Satisfaction with Life Scale

## References

[1] Hyder MA, Razzak J (2020). Telemedicine in the United States: An Introduction for Students and Residents. J Med Internet Res.

[2] Zaresani A, Scott A (2020). Does digital health technology improve physicians' job satisfaction and work-life balance? A cross-sectional national survey and regression analysis using an instrumental variable. BMJ Open.

[3] Elsner Peter (2020). Teledermatology in the times of COVID-19 - a systematic review. J Dtsch Dermatol Ges.

[4] Kayser M, Valtin C, Greer M, Karow B, Fuge J, Gottlieb J (2021). Video Consultation During the COVID-19 Pandemic: A Single Center's Experience with Lung Transplant Recipients. Telemed J E Health.

[5] Petersen W, Karpinski K, Backhaus L, Bierke S, Häner Martin (2021). A systematic review about telemedicine in orthopedics. Arch Orthop Trauma Surg.

[6] Gerbutavicius R, Brandlhuber U, Glück S, Kortüm G F, Kortüm I, Navarrete Orozco R, Rakitin M, Strodtbeck M, Wolf A, Kortüm K U (2021). Evaluation of patient satisfaction with an ophthalmology video consultation during the COVID-19 pandemic. Ophthalmologe.

[7] Gesamtzahl der Ärzte. Bundesärztekammer.

[8] Bundesärztekammer Berufstätige Ärzte.

[9] Statistisches Bundesamt Number of psychotherapists up 19% between 2015 and 2019. Statistisches Bundesamt.

[10] PhysioDeutschland Zahlen, Daten, Fakten zur Physiotherapie. PhysioDeutschland.

[11] Deutscher Verband der Ergotherapeuten e.V. (2017). Die Ergotherapie in Deutschland.

[12] Kassenärztliche Bundesvereinigung Immer mehr Praxen greifen zur Kamera - Zahl der Videosprechstunden auf über eine Million gestiegen. Kassenärztliche Bundesvereinigung.

[13] Allensbach ifD (2010). Ergebnisse einer Repräsentativbefragung von niedergelassenen und Krankenhausärzten im April/Mai 2010. In. Allensbach am Bodensee.

[14] Welle K, Täger Stefan, Hackenberg RK, Markowetz A, Schildberg FA, Burger C, Wirtz DC, Jansen T, Kabir K (2021). Examining the Hand in the Video Consultation. Z Orthop Unfall.

[15] Neller K (2005). Kooperation und verweigerung: eine non-response-studie [Co-operation and refusal: a non-response study]. ZUMA Nachrichten.

[16] Pepinsky TB (2018). A Note on Listwise Deletion versus Multiple Imputation. Polit. Anal.

[17] Klaus D, Mahne K, Wolff JK, Simonson J, Tesch-Römer C (2016). Daten und Methoden des Deutschen Alterssurveys. Altern im Wandel: Zwei Jahrzehnte Deutscher Alterssurvey (DEAS).

[18] UNESCO (2006). International Standard Classification of Education. ISCED 1997, Re-edition.

[19] Andresen EM, Malmgren JA, Carter WB, Patrick DL (1994). Screening for Depression in Well Older Adults: Evaluation of a Short Form of the CES-D. American Journal of Preventive Medicine.

[20] Mohebbi M, Nguyen V, McNeil JJ, Woods RL, Nelson MR, Shah RC, Storey E, Murray AM, Reid CM, Kirpach B, Wolfe R, Lockery JE, Berk M, ASPREE Investigator Group (2018). Psychometric properties of a short form of the Center for Epidemiologic Studies Depression (CES-D-10) scale for screening depressive symptoms in healthy community dwelling older adults. Gen Hosp Psychiatry.

[21] Diener E, Emmons RA, Larsen RJ, Griffin S (1985). The Satisfaction With Life Scale. J Pers Assess.

[22] Gierveld JDJ, Tilburg TV (2016). A 6-Item Scale for Overall, Emotional, and Social Loneliness. Res Aging.

[23] De Jong Gierveld J, Van Tilburg T (2010). The De Jong Gierveld short scales for emotional and social loneliness: tested on data from 7 countries in the UN generations and gender surveys. Eur J Ageing.

[24] Pavot W, Diener E, Colvin CR, Sandvik E (1991). Further validation of the Satisfaction with Life Scale: evidence for the cross-method convergence of well-being measures. J Pers Assess.

[25] Scott A, Bai T, Zhang Y (2021). Association between telehealth use and general practitioner characteristics during COVID-19: findings from a nationally representative survey of Australian doctors. BMJ Open.

[26] LaRoche KJ, Jozkowski KN, Crawford BL, Haus KR (2021). Attitudes of US adults toward using telemedicine to prescribe medication abortion during COVID-19: A mixed methods study. Contraception.

[27] Reitzle L, Schmidt C, Färber Francesca, Huebl L, Wieler LH, Ziese T, Heidemann C (2021). Perceived Access to Health Care Services and Relevance of Telemedicine during the COVID-19 Pandemic in Germany. Int J Environ Res Public Health.

[28] Wosik J, Fudim M, Cameron B, Gellad Z, Cho A, Phinney D, Curtis S, Roman M, Poon E, Ferranti J, Katz Jason N, Tcheng James (2020). Telehealth transformation: COVID-19 and the rise of virtual care. J Am Med Inform Assoc.

[29] HIMSS (2021). e-Health Trendbarometer - Consumer-enabled and Connected Health in Europe.

[30] Eberly LA, Kallan MJ, Julien HM, Haynes N, Khatana SAM, Nathan AS, Snider C, Chokshi NP, Eneanya ND, Takvorian SU, Anastos-Wallen R, Chaiyachati K, Ambrose M, O'Quinn Rupal, Seigerman M, Goldberg LR, Leri D, Choi K, Gitelman Y, Kolansky DM, Cappola TP, Ferrari VA, Hanson CW, Deleener ME, Adusumalli S (2020). Patient Characteristics Associated With Telemedicine Access for Primary and Specialty Ambulatory Care During the COVID-19 Pandemic. JAMA Netw Open.

[31] Sørensen Jens F L (2008). Attitudes toward telehealth use among rural residents: a Danish survey. J Rural Health.

[32] Hajek A, Bock J, König Hans-Helmut (2017). The role of personality in health care use: Results of a population-based longitudinal study in Germany. PLoS One.

[33] Agarwal R, Prasad J (1999). Are Individual Differences Germane to the Acceptance of New Information Technologies?. Decision Sciences.

[34] Burns A, Leavey G, Ward M, O’Sullivan R (2020). The impact of loneliness on healthcare use in older people: evidence from a nationally representative cohort. J Public Health (Berl.).

[35] Hajek A, Brettschneider C, Eisele M, Lühmann Dagmar, Mamone S, Wiese B, Weyerer S, Werle J, Fuchs A, Pentzek M, Stein J, Luck T, Weeg D, Mösch Edelgard, Heser K, Wagner M, Scherer M, Maier W, Riedel-Heller Steffi G, König Hans-Helmut, AgeCoDeAgeQualiDe Study Group (2019). Does transpersonal trust moderate the association between chronic conditions and general practitioner visits in the oldest old? Results of the AgeCoDe and AgeQualiDe study. Geriatr Gerontol Int.

[36] Kim ES, Kubzansky LD, Smith J (2015). Life satisfaction and use of preventive health care services. Health Psychol.

[37] George LS, Park CL (2013). Are meaning and purpose distinct? An examination of correlates and predictors. The Journal of Positive Psychology.

[38] Karataş Z, Uzun K, Tagay (2021). Relationships Between the Life Satisfaction, Meaning in Life, Hope and COVID-19 Fear for Turkish Adults During the COVID-19 Outbreak. Front Psychol.

[39] Hajek A, König Hans-Helmut (2019). Meaning in life and health care use: findings from a nationally representative study of older adults in Germany. BMC Geriatr.

[40] Kim ES, Strecher VJ, Ryff CD (2014). Purpose in life and use of preventive health care services. Proc Natl Acad Sci U S A.

[41] Klaus D, Engstler H, Mahne K, Wolff J, Simonson J, Wurm S, Tesch-Römer Clemens (2017). Cohort Profile: The German Ageing Survey (DEAS). Int J Epidemiol.

[42] Kane CK, Gillis K (2018). The Use Of Telemedicine By Physicians: Still The Exception Rather Than The Rule. Health Aff (Millwood).

[43] Jetty A, Moore MA, Coffman M, Petterson S, Bazemore A (2018). Rural Family Physicians Are Twice as Likely to Use Telehealth as Urban Family Physicians. Telemed J E Health.

